# Rheumatoid arthritis-associated cervical spine deformity and flares in disease activity

**DOI:** 10.1016/j.bas.2025.104235

**Published:** 2025-03-14

**Authors:** Anna B. Lebouille-Veldman, Dylan Spenkelink, Tom W.J. Huizinga, Carmen L.A. Vleggeert-Lankamp

**Affiliations:** a Department of Neurosurgery, Leiden University Medical Centre, Leiden, the Netherlands; b Department of Rheumatology, Leiden University Medical Centre, Leiden, the Netherlands; c Biomedical Engineering, University of Twente, the Netherlands; d Department of Neurosurgery, The Hague Medical Centre and HAGA Teaching Hospital, The Hague, the Netherlands; e Department of Neurosurgery, Spaarne Hospital Haarlem/Hoofddorp, the Netherlands

**Keywords:** Disease activity, Atlantoaxial subluxation, Vertical translocation, Rheumatoid arthritis, Cervical spine deformity

## Abstract

**Introduction:**

While rheumatoid arthritis (RA)-associated cervical spine deformity seems to be less prevalent following the introduction of the medication regimen to suppress inflammation in RA in an early stage, identifying patients at risk for atlantoaxial subluxation (AAS) or subaxial subluxation (SAS) remains challenging.

**Research question:**

The aim of the current study is to evaluate the association of the frequency of flares in systemic disease activity (DAS) and RA-associated cervical spine deformity.

**Materials and methods:**

This is a sub-analysis of the BeSt Study, where patients were treated to target DAS≤2.4. Lateral X-rays at 5- and 10-years follow-up were assessed for AAS and SAS.

**Results:**

Of 272 RA patients with radiographs of cervical spine that were included, 108 (40 %) had cervical deformity (AAS and/or SAS >2 mm). Although the number of patients with 3 or more flares was low, the majority of these patients did not demonstrate cervical spine deformity. After adjustment for the potential confounders age, gender, ACPA-status and RF-status, the presence of 3 or more flares was associated with a non-significant OR of 0.338 (95 % CI: 0.095–1.207) for the presence of RA-associated cervical spine deformity after 10 years.

**Discussion and conclusion:**

A trend towards less RA-associated cervical deformity in patients with more flares was discerned, though no statistically significant differences could be established. It is hypothesized that the occurrence of a flare leads to an increase in medication, which may in turn protect the cervical spine from developing deformities. Future studies should more in detail explore the effect of medication on cervical deformity.

## Highlights

• In this group of closely monitored RA patients, flare ups of disease activity regularly occurred.
• RA treatment regimens may need to be aimed at more than just DAS values, to avoid cervical deformity in RA in the long term.
• This study did not show a significant association between number and duration of flares and RA-associated cervical spine deformity after long-term follow-up."

## 1 Introduction

Rheumatoid arthritis (RA) is a chronic inflammatory disease, which affects circa 1 % of the population (Goekoop-Ruiterman et al., 2008). In particular in patients with autoantibodies such as anti-citrullinated protein antibodies (ACPA), chronic inflammation leads to damage to joint structures and loss of joint integrity (van den et al., 2012). Hands and feet are most commonly involved in RA, but the cervical spine can also be affected.

The cervical vertebrae are stabilized by joints, intervertebral discs, and an intricate network of ligaments. Rheumatoid Arthritis can affect these ligaments and cause laxity, which in turn can lead to subluxation of vertebral bodies and instability. Characteristically, cervical spine damage is scored using three radiological deformity parameters: 1) in the upper cervical region, subluxation of the C1-C2 vertebra causes atlantoaxial subluxation (AAS). This subluxation may be accompanied by occipital pain caused by compression of the major occipital nerve, which is the medial branch of the dorsal primary ramus of the cervical spinal nerve C2 that arises between the first and second cervical vertebrae; 2) a severe form of atlantoaxial subluxation, which is usually accompanied by erosion of the odontoid peg, is referred to as ‘vertical translocation’ (VT) (Kauppi et al., 2005), and 3) below the upper cervical deformities, subaxial subluxation (SAS) is a common phenomenon in the cervical spine of RA patients. Mild forms of cervical spine deformations can be asymptomatic, but subluxations can lead to compression of the spinal cord and medulla oblongata, which can cause severe neurological deficits and even sudden death. It is generally believed that once neurological deficits occur, progression is inevitable, although rapidity of progression is highly variable (Goekoop-Ruiterman et al., 2008).

In previous years, the cervical spine was reported to be affected in variable frequencies in RA (17–86 % of RA patients)(Moskovich et al., 2000), but with current RA medical treatment policies, clinically relevant involvement of the cervical spine seems to have declined. Current RA medical treatment is aiming at lowering systemic inflammatory activity (DAS; Disease Activity Score). It would therefore be logical if this decrease in DAS would correlate to a decline in the prevalence of cervical spine deformity. We conducted a systematic review of literature and demonstrated that conclusions on the association between systemic disease activity and cervical deformity varied widely (Veldman et al., 2022).

We subsequently evaluated our own data for the association of cervical deformity and systemic disease activity in RA patients (Lebouille-Veldman et al., 2023). A cohort of 272 newly onset RA patients were treated to target low disease activity (Disease Activity Score DAS≤2.4) with varying treatment protocols. Every three months DAS was assessed and medication was adjusted according to protocol to induce lowering of DAS scores. If DAS scores decreased below 1.6 (remission) and this score maintained below this value for six months (sustained remission), medication was stopped. In this trial up to 50 % of patients achieved remission and up to 29 % patients reached drug free remission. However, some patients never reached remission or low DAS (Allaart et al., 2018).

To study the association of DAS values and cervical deformity we studied the prevalence of deformity after 10 years follow up. Despite the targeted treatment, still 3 % of patients developed AAS ≥5 mm in flexion and 24 % developed AAS ≥3 mm in flexion. No definite association could be observed between mean disease activity score or sustained remission after 10 years follow up, and the presence or absence of cervical spine deformity after 10 years follow-up (Lebouille-Veldman et al., 2023).

As the disease activity in patients with RA has been demonstrated to vary considerably throughout the course of the disease (Veldman et al., 2022; Lebouille-Veldman et al., 2023), prevalence of cervical spine deformity might be correlated to flares in disease activity. In a sub-analysis of the BeSt study, it was previously established that patients who had multiple episodes of increase in disease activity demonstrated an increased risk for development of radiological damage to the joints of hands and feet (Markusse et al., 2015). This study aims to study if an association between flares in disease activity and cervical spine deformity can be established.

## 2 Materials and methods

This study uses data from the BeSt trial which is a single-blinded multicenter randomized trial, designed to compare four treatment strategies in patients recently diagnosed with active RA (all then fulfilling the American College of Rheumatology, 1987 classification criteria), with at least 6 inflamed joints (of 66 assessed) and either a high erythrocyte sedimentation rate or a high patient rating of disease activity (Markusse et al., 2016). Patients were recruited in 18 non-university and 2 university hospitals in The Netherlands between 2000 and 2002. The Medical Ethics Committee of the LUMC approved the study (P08.011) and the regulatory boards of the individual hospitals approved likewise. All patients gave written informed consent. Adherence to the STROBE statement guidelines was ensured in both the conduct and reporting of the study (von Elm et al., 2007).

Patients were randomized to the following treatment arms: (1) sequential monotherapy (starting with methotrexate monotherapy); (2) step-up combination therapy (also starting with methotrexate monotherapy); (3) initial combination therapy with methotrexate, sulfasalazine and prednisone; and (4) initial combination therapy with methotrexate and infliximab. All 508 patients were treated according to the ‘treat to target’ principle, requiring protocolized treatment adjustments based on three-monthly assessments of the Disease Activity Score (DAS; based on a 44 (for swelling)/53 (for tenderness using the Ritchie Articular Index) joint count, ESR and patient's assessment of disease activity) (Salaffi et al., 2021).

In case of a DAS >2.4, treatment was increased according to the next step in the relevant treatment strategy arm. In case of DAS≤2.4 for at least 6 consecutive months, treatment was stepwise tapered to low dose monotherapy, and if subsequently remission (DAS<1.6) was achieved for at least 6 months, all treatment was stopped (drug free remission), but restarted if DAS later increased to ≥1.6 (Markusse et al., 2016).

### 2.1 Assessment of RA-associated cervical spine deformity

No baseline cervical spine radiographs were available. Lateral X-rays of the cervical spine were collected at 5- and 10-years follow-up.

Radiological cervical deformity parameters (AAS, SAS and VT) were evaluated on lateral X-rays by two researchers (ABV and CVL), both blinded for patient characteristics (Lebouille-Veldman et al., 2023). Agreement was reached in close cooperation. If a dynamic X-ray was performed (flexion/extension), AAS was scored separately on these radiographs.

Cervical spine deformity was defined as the presence of AAS, defined as a distance >2 mm between the odontoid peg and the anterior arch of C1 in neutral position and/or the presence of SAS. AAS of ≥3 mm in flexion on dynamic X-ray was a secondary outcome. Severe AAS was defined as AAS of ≥5 mm in flexion or neutral position. SAS was concluded to be present in case a listhesis of more 2 mm existed in neutral position. VT was present if the tip of the odontoid peg exceeded the line of McGregor (Kim and Hilibrand, 2005).

If an X-ray was missing at 10 years follow-up and AAS, VT or SAS was present at 5 years, it was scored to be also present at 10 years follow-up. For the AAS of ≥3 mm in flexion and severe AAS groups, if a flexion X-ray was missing at 10 years of follow-up and AAS was not present at 5 years, the patient was not included in the sub analysis for these groups.

### 2.2 Definition of flare

During a period of 10 years, DAS was measured every 3 months, and thus 41 times in total. The DAS endpoint was assessed using the DAS value at 10 years, where a value of <1.60 was considered remission. Sustained remission was reached when DAS <1.60 for ≥6 months. Remission is an important endpoint in this study, as it indicates the possibility to stop treatment (drug-free remission) (Markusse et al., 2016).

Tapering of treatment with medication was started once DAS was ≤2.4. DAS was evaluated every 3 months and medication dose was increased if DAS was above 2.4. If sustained remission (DAS<1.60 for ≥6 months) was reached, medication was stopped. However, when DAS flared above 1.60 medication was started again (Markusse et al., 2016). This was defined as a flare in DAS in this study.

The presence or absence of sustained remission at each time point during 10 years follow-up (41 time points) was evaluated, by checking whether at the 3 preceding time points (covering 6 months' time) DAS was below 1.60. Once sustained remission at a certain timepoint was obtained, it was evaluated whether DAS exceeded the 1.60 value again, and this was scored as a ‘flare’.

The current definition of flares differs from the definition set by Markusse et al., where a flare was counted if DAS was higher than 2.4 with an increase of ≥0.6 regardless of previous DAS (Markusse et al., 2015). The current definition has a clinical implication as it represents a restart of treatment after previous tapering and/or discontinuation of treatment; hence it was decided to use this definition in the present study.

Since a flare in the current definition leads to intensify treatment, it is expected that disease activity will lower after some time. Therefore, it is of interest how long a flare lasts. We thus introduce the term ‘cumulative duration of flares’. This is the sum of the total number of months that a patient experienced flares.

### 2.3 Statistical analysis

Baseline data were expressed as mean ± SD or number ( %) and compared using *t*-test analysis.

Multiple imputation was used for missing DAS values. The imputation model included terms for treatment strategy, age, gender, ACPA status, Rheumatoid Factor status, HAQ and DAS values. In the imputation, 20 iterations were pooled using MATLAB 2019b and combined to form the individual DAS data that were used to explore correlations, and also to form a mean DAS value.

For evaluation of associations in cervical deformity, binomial logistic regression was performed to study the association with *number of flares*. This model was corrected for the possibly confounding factors age, gender, ACPA-status and RF-status. The number of flares was categorized to represent four groups: no flares (always in sustained remission); 1–2 flares; ≥3 flares; 0 flares (also defined as ‘continuous flare’, since these patients never reached sustained remission). Binary logistic regression was also performed to study the association between cervical deformity and the *cumulative duration of flares*. Likewise, this model was corrected for the possibly confounding factors age, gender, ACPA-status and RF-status.

Statistical analyses were performed using SPSS version 29.1 and MATLAB version 2019b (IBM Corp. Released, 2021; Inc. TM).

## 3 Results

331 of 508 patients completed 10 years of clinical follow-up. 20 patients were excluded as they were missing both X-ray images at 5 and 10 years of follow-up. Additionally, 39 patients were excluded because they were missing the X-ray at 10 years and had no signs of cervical deformity at 5 years follow-up. Eventually 272 patients had adequate radiological (neutral radiographs at 10 years follow-up present, or cervical deformity present in the 5-year follow-up neutral radiographs) and DAS follow up data.

After 10 years, 62 of 272 patients (23 %) had Atlantoaxial subluxation (AAS) of more than 2 mm in neutral position. 60 of 272 patients (22 %) had subaxial subluxation (SAS). No patients had VT above the line of McGregor. In total, 108 of 272 patients (40 %) had cervical spine deformity of any kind on neutral X-ray. In this group of patients, the mean age at baseline was 55.2 ± 12.7 years, 60 % was female, 69 % of patients were RF positive and 68 % were ACPA positive. This was comparable to the demographic data of the patients without cervical spine deformity, except for age. Patients with cervical spine deformity after 10 years were significantly older than patients without cervical spine deformity (50.6 (±11.2) years; p = 0.002; Table 1).

**Table 1:** Baseline characteristics An overview is provided of the differences between the groups of patients with and without cervical spine deformity at baseline. Cervical spine deformity in defined as AAS and/or SAS of >2 mm.

Characteristic	Cervical spine deformity at 10 years follow-up (n = 108)	No cervical spine deformity at 10 years follow-up (n = 164)
**Mean age at baseline (SD)**	55.2 (±12.7)	50.6 (±11.2)
**Female, n ( %)**	65 (60 %)	117 (71 %)
**Mean DAS at baseline (SD)**	4.39 (±0.89)	4.34 (±0.86)
**Mean HAQ score at baseline (SD)**	1.22 (±0.66)	1.36 (±0.63)
**RF-positive, n ( %)**	74 (69 %)	112 (68 %)
**ACPA-positive, n ( %)**	73 (68 %)	104 (63 %)

Abbreviations: DAS: Disease Activity Score; HAQ: Health Assessment Questionnaire; RF: rheumatoid factor; ACPA: anti-citrullinated protein antibodies.

109 patients had an X-ray in flexion available. 26 patients (24 %) of these demonstrated AAS ≥3 mm in flexion. 2 of these patients demonstrated AAS ≥5 mm in flexion. Additionally, on the neutral X-rays 6 patients demonstrated an AAS ≥5 mm. Consequently, a total of 8 patients (3 %) had AAS ≥5 mm in flexion and/or neutral position (severe AAS). (Fig. 3)

### 3.1 Disease activity and flares

At baseline, mean DAS was 4.39 (±0.89) in the group of patients with cervical spine deformity and 4.33 (±0.86) in the group of patients without cervical spine deformity. After 10 years of follow-up, 135 (50 %) of patients were in remission (DAS<1.60). 87 (32 %) patients reached sustained remission at 10 years follow-up (DAS<1.60 for 6 months).

**Fig. 1:**
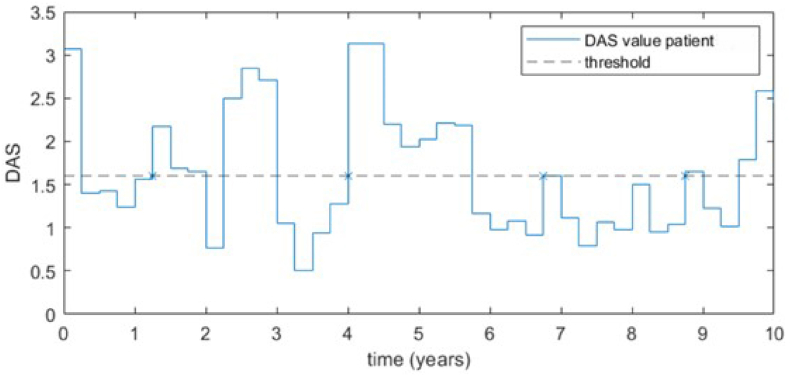
**DAS values of patient in the BeSt Trial during 10 years of follow-up.** In this figure, the variability of disease activity in one of the patients in the trial is depicted. It shows a line at 1.60. If a patient had a DAS ≤1.60 during ≥6 months, the patient would be in sustained remission. However, a flare occurred once the DAS of the patient went above 1.60 again after this period of sustained remission. Flares are indicated by crosses on the 1.60 line for this patient.

Patients who did not achieve sustained remission at any time during follow-up did, by definition, not demonstrate a flare as defined for this study. Seventy-two percent of patients with and 78 % of patients without cervical spine deformity reached sustained remission at least once during 10 years follow-up, and in some patients disease activity varied largely and they thus demonstrated several flares. An example of the course of DAS in a patient demonstrating several flares, is depicted in Fig. 1.

The number of patients that never had an increase of DAS above a value of 1.60 (after the decrease of the baseline DAS), indicated as ‘always in sustained remission’, was very low. The majority of patients had 1-2 flares, equally divided over the number of 1 and 2 flares during the 10 years follow up period. A minority of patients had 3 or more flares.

The mean cumulative duration of flares in the group of patients with cervical spine deformity after 10 years follow-up was 66 ± 43 months, compared to 62 ± 41 months in the group of patients without cervical spine deformity after 10 years.

### 3.2 Association between RA-associated cervical spine deformity and number of flares

**Fig. 2:**
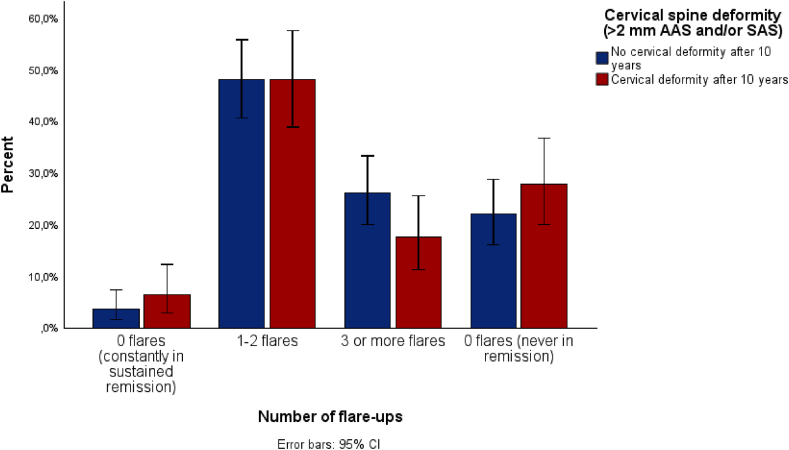
**Percentage of patients experiencing a certain number of flares during 10 years follow-up.** This bar chart depicts the percentage of patients experiencing a certain number of flares during 10 years follow-up.

In comparing the number of flares between patients with and without cervical spine deformity, no statistically significant differences between the two groups of patients were demonstrated (Table 2). However, although the number of patients with 3 or more flares was low, the majority of these patients did not demonstrate cervical spine deformity (Fig. 2). In contrast, in the patients that never reached sustained remission, the number of flares were equally divided over the patients with and without cervical spine deformity (Table 2).

**Table 2:** Comparing number of flares per patient to the presence of cervical deformity after 10 years follow-up This table shows the number of flares categorized in four categories, compared to the presence of cervical deformity after 10 years follow-up according to multiple definitions.

Number of flares	AAS and/or SAS >2 mm (n = 272)		AAS ≥3 mm in flexion (n = 109)		AAS ≥5 mm (n = 107)	
Univariate	Present after 10 years (n = 108)	Absent after 10 years (n = 164)	Present after 10 years (n = 41)	Absent after 10 years (n = 82)	Present after 10 years (n = 8)	Absent after 10 years (n = 99)
**0 flares (always in sustained remission)**	7 (6.5 %)	6 (3.7 %)	2 (7.7 %)	1 (1.2 %)	0	3 (3.0 %)
**1**–**2 flares**	52 (48.1 %)	79 (48.2 %)	15 (57.7 %)	35 (42.2 %)	5 (62.5 %)	43 (43.4 %)
***1 flare***	*29 (27 %)*	*44 (27 %)*	*8 (20 %)*	*21 (26 %)*	*1 (13 %)*	*27 (27 %)*
***2 flares***	*23(21 %)*	*35 (21 %)*	*7 (17 %)*	*14 (17 %)*	*4 (50 %)*	*16 (16 %)*
**3 or more flares**	19 (17.6 %)	43 (26.2 %)	5 (19.2 %)	24 (28.9 %)	1 (12.5 %)	28 (28.3 %)
**0 flares (never in sustained remission)**	30 (27.8 %)	36 (22.0 %)	4 (15.4 %)	23 (27.7 %)	2 (25.0 %)	25 (25.3 %)

**P-value**	0.240		0.115∗		0.667∗	

Note: P-value determined by chi square test (Fisher exact test). ∗Not all expected counts were more than 5.

Abbreviations: DAS: Disease Activity Score; AAS: Atlantoaxial Subluxation; SAS: subaxial subluxation.

**Fig. 3:**
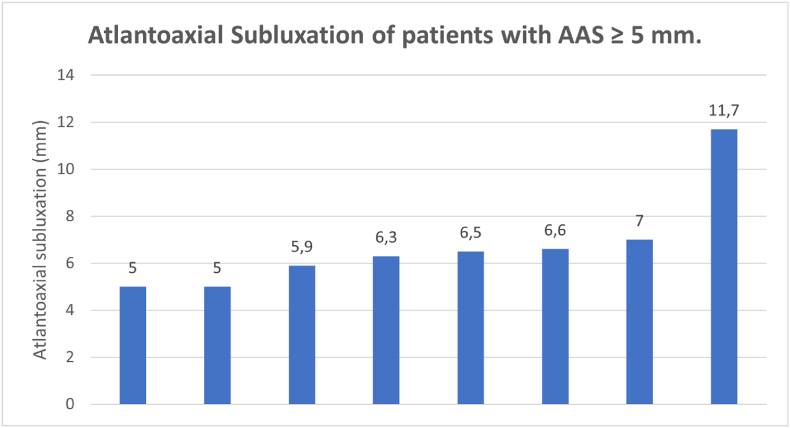
**Atlantoaxial Subluxation of patients with AAS ≥ 5 mm.** This figure shows the exact measure (in mm) of AAS for the 8 patients who had AAS of 5 mm or more on flexion or neutral X-rays.

After adjustment for the potential confounders age, gender, ACPA-status and RF-status, the presence of 3 or more flares was associated with an OR of 0.338 (95 % CI: 0.095–1.207) for developing cervical spine deformity after 10 years (Table 3). For the patients with 1–2 flares the OR was 0.545 (95 % CI: 0.164–1.811) and for patients who never reached sustained remission the OR was 0.770 (CI 95 %: 0.217–2.731).

**Table 3:** Binomial logistic regression for number of flares (for cervical spine deformity at 10 years follow-up (AAS >2 mm and/or SAS >2 mm) This table shows the results of a binomial logistic regression performed to further evaluate the association between cervical spine deformity after 10 years follow-up and the number of flares per patient.

Variable	Number of patients	Odds Ratio^a^	P^a^	95 % Confidence Interval for OR^a^	
				Lower	Upper
**Number of flares**					
**0 flares (always in sustained remission)**	13		0.130		
**1**–**2 flares**	131	0.545	0.322	0.164	1.811
**≥3 flares**	62	0.338	0.095	0.095	1.207
**0 flares (never in sustained remission)**	66	0.770	0.686	0.217	2.731

Abbreviations: DAS: Disease Activity Score; AAS: Atlantoaxial Subluxation; SAS: subaxial subluxation.

a Corrected for age at baseline, gender (male or female), anti-CCP positivity and Rheumatoid Factor positivity.

After adjustment for the potential confounders age, gender, ACPA-status and RF-status, the total duration of flares was associated with an OR of 1.008 (95 % CI 0.989–1.027) for developing cervical spine deformity after 10 years.

## 4 Discussion

In a cohort of early onset RA patients, in whom medication was carefully adjusted upon changes in disease activity, a close association between systemic activity and deformity is expected. However, in a previous paper we demonstrated the absence of an association between the mean systemic disease activity (DAS) over a ten year follow up period and the presence of cervical spine deformity, as well as the absence of an association between reaching remission after ten years and the presence of cervical deformity (Lebouille-Veldman et al., 2023).

In the current study, in which we hypothesized that the variability in DAS in RA patients played a role in the development of cervical deformity, no unequivocal association between flares in systemic disease activity and deformity could be demonstrated either. However, there was a trend towards a lower odds of cervical spine deformity in patients with 3 or more flares. This is remarkable and contra-intuitive, since it would be expected that the patients with cervical spine deformity would have experienced more flares and not less. A possible explanation is that the medication regimen is intensified at every time DAS flares up. Along that line of reasoning, patients with more flares are more frequently subjected to intensified anti-rheumatic medication.

This explanation is, however, contradicted by the observation that in the group of patients who never reached sustained remission, the occurrence of cervical spine deformity was more frequent.

It can be concluded though, that the current results confirm the absence of an association between DAS and the development of cervical spine deformity. This confirms the statement that RA medication treatment regimens should not be solely aimed at DAS values, as they are currently defined, in order to avoid cervical deformity in RA in the long term.

Our results imply that it is not recommendable to stop anti-rheumatic medication if treatment has the purpose to prevent cervical deformity. The medication to treat the inflammation in RA, however, also may have side-effects and this is the rationale to withdraw medication in periods of continuous remission. Ideally, a prediction model can be developed to indicate which patient is prone to develop cervical deformity, in order to personalize the medication regimen. In developing such a prediction model, more parameters should be considered than merely the factors that define the DAS.

The definitions we applied to evaluate AAS, SAS and VT are commonly used, but several variations are possible. Since there is much discussion on the most appropriate definition for AAS, several methods were applied to evaluate AAS. The distance between atlas and axis of 5 mm in flexion is the most convincing parameter that a pathological relation exists. We consider it the most striking result that in a cohort of patients with newly onset RA, in which their treating physicians monitored the disease activity carefully and treated it optimally, still 3 % of patients developed severe AAS. Moreover, it is remarkable that there was no trend of any kind that in this group of patients control of disease activity was worse compared to patients without severe AAS.

There are several limitations to this study. First, it would have been optimal if we would have had X-rays in flexion, extension and neutral position of all patients at baseline, 5- and 10-years follow-up. Secondly, X-rays are in some cases challenging to interpret and quantify. The X-rays were made in the workflow of a study aiming at clinical parameters and the current radiological evaluations were not done instantly. In daily practice, if an X-ray of the cervical spine in RA patients is performed, it is evaluated carefully and, if difficult to interpret, made again. This process would have yielded more qualitative X-rays in some cases, and a better follow up over the years. Furthermore, particularly excluding the patients missing the 10 years follow up X-ray after their 5-year X-ray demonstrated no deformity, is possibly inducing some selection bias. Although this was relatively rare, it could lead to underestimation of the true presence of deformity.

In future studies, the actual given medication should be evaluated to study the true effect of medication and determine the best strategy to prevent cervical spine deformity, if possible.

**In conclusion, this study did not show a significant association between number and duration of flares and RA-associated cervical spine deformity after long-term** follow-up**. This** raises the hypothesis that RA medication treatment regimens should not be solely aimed at DAS values, as they are currently defined, in order to avoid cervical deformity in RA in the long term.

## Funding

This research did not receive any specific grant from funding agencies in the public, commercial, or not-for-profit sectors.

## Declaration of competing interest

The authors declare that they have no known competing financial interests or personal relationships that could have appeared to influence the work reported in this paper.

## Appendix A Supplementary data

Supplemental TABLE 1: In- and exclusion criteria.

This supplemental table depicts the in- and exclusion criteria of the BeSt Trial (Lebouille-Veldman et al., 2023; Markusse et al., 2016).
