# Supplementary material for: Rheumatoid arthritis-associated cervical spine deformity and flares in disease activity
Source: Brain Spine. 2025 Mar 14;5:104235. doi: 10.1016/j.bas.2025.104235 (PMC11979421; doi:10.1016/j.bas.2025.104235)
Supplement: Multimedia component 1 [file mmc1.docx]

**Supplemental table 1: In- and exclusion criteria**

| Inclusion criteria In order to be eligible to participate in this study, a subject must meet all of the following criteria:   - Availability of lateral cervical X-ray at 5 years and 10 years of follow-up - 18 years or older - Diagnosed with RA; They have active disease with 6 or more swollen joints and 6 or more painful joints and at least one of the following:   1. Westergren erythrocyte sedimentation rate (ESR) of at least 28 mm/hour.  2. Patient’s global assessment of general well-being of at least 20 mm measured on a 100 mm horizontal visual analogue scale (VAS).   - Informed consent - 6 or more swollen joints - 6 or more painful joints - At least one of the following: - Westergren erythrocyte sedimentation rate (ESR) of at least 28 mm/hour. - Patient’s global assessment of their general well-being of at least 20 mm measured on a 100 mm horizontal visual analogue scale (VAS). |
| --- |
| Exclusion criteria A potential subject that meets any of the following criteria will be excluded from participation in this study:   - Previous therapy with DMARDs except for hydroxychloroquine - Pregnancy or wish to become pregnant during the study, or childbearing potential without adequate contraception - Concomitant treatment with another experimental drug - History or presence of malignancy within the last five years - Bone marrow hypoplasia - Elevated hepatic enzyme levels (ASAT, ALAT > 3 times normal value) - Serum creatinine level >150 umol/L or estimated creatinine clearance of <75 mL/ min - Diabetes mellitus - Alcohol or drug abuse |
